# Conscious sedation and dental treatment outcomes in Norwegian paediatric dentistry: a retrospective clinical audit from the Public Dental Service

**DOI:** 10.1007/s40368-026-01178-y

**Published:** 2026-02-08

**Authors:** Hege Nermo, Emilie Sofie Apelqvist, Emilie Johansen, K Nordmo, Elin Synnøve Hadler-Olsen

**Affiliations:** 1 The Public Dental Health Competence Center of Northern Norway, Tromsø, Norway; 2 Department of Clinical Dentistry, Faculty of Health Sciences, UiT The Arctic University of Norway, Tromsø, Norway; 3 Department of Medical Biology, Faculty of Health Sciences, UiT The Arctic University of Norway, Tromsø, Norway

**Keywords:** Conscious sedation, Paediatric dentistry, Public dental service, Behaviour management, Restraint, Dental anxiety

## Abstract

**Purpose:**

Conscious sedation is recommended in paediatric dentistry to alleviate distress, provided it complements behavioural management, avoids restraint, and includes structured follow-up. Its use in the Norwegian Public Dental Service (PDS) has not been systematically described. This study aimed to characterise sedation practices in the PDS and examine factors associated with dental treatment failure in children receiving oral conscious sedation.

**Methods:**

A retrospective audit was conducted in three PDS clinics (August 2022–July 2023), analysing 201 sedation sessions for patients aged 0–18 years. Indications, challenges, outcomes, and follow-up were coded from records. Logistic regression was used to assess variables associated with dental treatment failure, defined as incomplete or omitted treatment.

**Results:**

Children aged 6–10 years accounted for 54.7% of sessions, while preschoolers accounted for 26.9%. Behavioural management problems (BMP) were common in younger children, while dental fear/anxiety predominated in older groups. Restraint occurred in 16% of cases, and follow-up was documented in 21%. Treatment failure occurred in 32.3% of sessions. In adjusted analyses, both BMP (odds ratio [OR] 1.97, 95% confidence interval [CI] 1.01–4.08) and physical restraint (OR 3.95, 95% CI 1.84–10.22) were significantly associated with treatment failure. No serious adverse events were recorded.

**Conclusions:**

Oral conscious sedation is widely used and appears safe in the immediate clinical setting. However, one-third of sessions resulted in incomplete treatment, with failure more frequently observed in sessions where BMP were recorded as the indication for sedation, and in sessions where physical restraint occurred. These findings underscore the importance of careful indication, case selection, and follow-up beyond procedural completion to support child-centred outcomes.

## Introduction

Norwegian children benefit from a comprehensive and proactive public dental service (PDS), which is provided free of charge up to the age of 18 (Ministry of Health and Care Services 1984). From the age of 3, children are routinely recalled for preventive and restorative care. Combined with widespread fluoride use, this is believed to have contributed to a marked decline in caries (Statistics Norway 2023). While most children today require little more than preventive visits, a minority develop extensive disease. For some, treatment cooperation cannot be achieved through behavioural management alone. In such cases, conscious sedation may be considered to facilitate treatment by reducing overt distress and discomfort.

International and national guidelines emphasise that sedation should support, not replace, behavioural strategies, that restraint should be avoided, and that structured follow-up is essential to safeguard the child’s coping and trust (Ashley et al. 2021, Coté et al. 2019, Norwegian Directorate of Health 2024). However, evidence suggests that competence and confidence in behavioural management techniques (BMT) vary considerably, with a high demand for further training even among paediatric dental specialists (Wilson and Alcaino 2011). Consequently, the extent to which these guideline principles are consistently applied in everyday practice remains uncertain.

Children requiring sedation are not a homogeneous group. Caries is socially patterned, with higher prevalence among children from disadvantaged or immigrant backgrounds (Wigen and Wang 2014, 2010). Psychosocial adversities may further complicate coping with dental treatment, as adverse childhood experiences (ACEs), including neglect and maltreatment, are linked to both poorer oral health and dental fear (Myran et al. 2025; Kvist et al. 2018). These associations underline the importance of structured follow-up to prevent long-term anxiety and avoidance. Nevertheless, such vulnerabilities are not assessed or documented in routine practice, where decisions about sedation are usually based on observed behaviour and cooperation in the chair. This gap risks leaving vulnerable children’s needs unrecognised and unaddressed.

In the Norwegian Public Dental Service (PDS), paediatric care and conscious sedation are primarily provided by general practitioners, whose competence in BMT depends on undergraduate training and individual experience. National specialist capacity in paediatric dentistry is low, and only a small number of specialists work within the PDS. Access to nitrous oxide sedation is also uneven, as its use requires additional certification and appropriate ventilation infrastructure. Specialist services, nitrous oxide facilities, and general anaesthesia are all centralised, meaning that referral pathways are often constrained by long travel distances and waiting lists, which in turn shape treatment decisions in everyday care. This stands in contrast to several European countries, where inhalation sedation and specialist-delivered services are more widely available, while oral benzodiazepine sedation is used far less frequently. These contextual differences help explain why oral sedation remains relatively common in the Norwegian PDS.

Taken together, these factors underscore the crucial need to investigate not only how conscious sedation is applied in the Norwegian PDS, but also its impact on dental treatment outcomes. If sedation does not enable treatment completion, the considerable resources and potential risks of oversedation, procedural distress, or reliance on physical restraint may not be justified. Evidence gaps remain regarding the consistency of follow-up, the use of restraint, and the likelihood of successful treatment. Against this background, the present retrospective clinical audit aimed to map sedation practices in three Norwegian PDS clinics, focusing on indications, outcomes, procedural challenges, and follow-up. By situating the Norwegian experience within an international framework, the study seeks to clarify both opportunities and challenges in ensuring safe, equitable, and dignity-preserving dental care for children who struggle most to cope with treatment.

## Materials and methods

### Study design and setting

This was a retrospective observational clinical audit based on anonymised internal quality assurance data from three public dental clinics in Norway, representing different regions and serving both urban and rural populations. Clinics were selected as a convenience sample, as dental students in clinical training at these sites could facilitate data collection.

### Participants

All conscious sedation sessions involving patients aged 0–18 years, conducted between August 2022 and July 2023, were included in the analysis. Conscious sedation was defined as the planned use of oral sedative medication to facilitate dental treatment while maintaining protective reflexes and cooperation. The term is used deliberately to denote a level of sedation in which the child remains awake and responsive to verbal stimulation, in accordance with European paediatric dental guidelines. In accordance with the Norwegian national clinical guideline for the Public Dental Service (Norwegian Directorate of Health 2024), oral midazolam is recommended as first-line sedation in the dental clinic, with diazepam as an alternative. In routine clinical practice, oral midazolam is administered at a weight-based dose of 0.5 mg/kg body weight (maximum 12.5 mg), typically 30 min before treatment. An extemporaneously prepared oral mixture (2 mg/mL) based on the injectable formulation is commonly used, in accordance with national pharmacological recommendations (Norwegian Medicines Agency 2017). Combination or multi-drug sedation is not recommended in this setting. Although weight-based dosing of midazolam is routinely documented in the PDS, these fields were not included in the anonymised audit dataset and could therefore not be analysed. Nitrous oxide inhalation sedation was excluded because its use requires additional certification and specialised ventilation infrastructure and is therefore not uniformly available across clinics in the Norwegian Public Dental Service.

The three clinics together had responsibility for 20,119 children (0–18 years) during the study period. However, not all children are recalled annually; recall intervals are based on caries risk and developmental stage.

### Data collection and measures

Each clinic retrospectively compiled internal quality assurance data by pairing information from Class A and B medication logs, as required by Norwegian health legislation, with entries from electronic dental records. Based on these internal audits, predefined variables were aggregated into a standardised electronic questionnaire (Nettskjema.no, University of Oslo; approved for handling highly confidential data). Data entry was performed by personnel in clinical training who had received instruction and calibration in the use of predefined categories. Only anonymised variables approved by data protection officers were extracted for this audit; researchers had no access to identifiers or linkage keys.

### Variables and coding

Age group was categorised as 0–5, 6–10, 11–15, and 16–18 years.

Indications for sedation were classified into four categories. *Dental fear/anxiety (DFA)* referred to records where dental anxiety was noted, either explicitly as self-reported fear by the child or caregiver, or as defined by the clinician. *Behavioural management problems (BMP)* referred to records describing a lack of cooperation or disruptive/uncooperative behaviour despite attempts at desensitisation, including complete refusal to undergo treatment. If both DFA and BMP were documented in the record, both categories were coded; thus, indications were not mutually exclusive. *Acute* indicated treatment was performed due to urgent need, such as pain or trauma, without prior desensitisation or extensive treatment need. *Not specified* was used when no indication was documented in the record. The largest overlap was between DFA and BMP, which were recorded together in 12 sessions (6.0% of all sedation cases; 11.5% of those with either DFA or BMP documented). For descriptive analyses, all recorded indications were reported. In regression analyses, indications were not entered as separate explanatory variables; instead, BMP was tested as an explanatory variable alongside age and the use of physical restraint.

Treatment outcome was coded as *completed* (planned procedure finished as intended), *partial/suboptimal* (treatment performed but of compromised quality, e.g. incomplete caries removal, poor isolation, or temporary materials), or *not performed* (no operative treatment beyond local anaesthesia). For regression analyses, partial/suboptimal and not performed were combined into the outcome *dental treatment failure*, with completed treatment as the reference category.

Procedural challenges were classified as *physical restraint* (explicit documentation of active holding of the child’s head, hands, or body during treatment), *insufficient effect* (no observable calming effect or paradoxical reaction with increased agitation or resistance), *oversedation* (reported reduction in oxygen saturation or consciousness), *administration problems* (refusal, spitting out, or partial ingestion of the sedative), or *none*. Mechanical stabilisation devices (e.g. papoose boards), which involve planned mechanical immobilisation, are not used in the Norwegian Public Dental Service and were therefore not included. More than one challenge could be recorded for a single session. The most frequent overlap was between insufficient effect and physical restraint, which co-occurred in 10 sessions (5.0% of all sedation cases; 25% of cases with insufficient effect). In regression analyses, only physical restraint was entered as a covariate, as it was the only factor that could be documented unambiguously, whereas insufficient effect could not be reliably operationalised as a separate variable based on retrospective chart review.

Follow-up was recorded at two levels. General follow-up after sedation was coded as ‘none’, ‘desensitisation visit’, ‘shorter recall interval’, or ‘telephone contact’. Additional follow-up after incomplete or suboptimal treatment was coded as a new attempt within six months, a new attempt at subsequent recall (> 6 months), referral to another practitioner or paediatric dental specialist, referral for treatment under general anaesthesia, or not specified. At both levels, multiple categories could be selected if more than one form of follow-up was documented. In practice, however, overlap between categories was rare and is therefore described separately in the results.

Since most variables were derived from free-text entries in patient records, classification required interpretation by the auditing clinicians; only age and treatment outcome were available as structured fields.

### Ethics

The audit used only retrospective, anonymised data from quality assurance studies of routine clinical practice and introduced no additional burden for patients. This project aimed to evaluate existing practices in light of established guidelines, with the goal of improving or assessing current procedures. According to Norwegian regulations, such quality assurance studies are not subject to approval by the Regional Committees for Medical and Health Research Ethics (REK), as they do not aim to generate new, generalisable knowledge. Approval for the study was obtained from the county dental authorities and institutional data protection officers prior to data collection, and all procedures adhered to the Declaration of Helsinki.

### Statistical analysis

Descriptive statistics were used to summarise indications, outcomes, procedural challenges, and follow-up. Group differences were examined using chi-square tests (Pearson’s χ^2^ for tables with > 2 categories; χ^2^ with continuity correction for 2 × 2 tables). In addition to descriptive analyses, crude odds ratios (OR) with 95% confidence intervals (CI) were estimated for individual explanatory variables of dental treatment failure.

For multivariable analysis, a binary logistic regression model was fitted, with dental treatment failure (treatment partial/suboptimal or not performed) as the dependent variable versus treatment completed as planned. Based on theoretical relevance and data quality, three explanatory variables were included: (i) age group (11–18 vs. 0–10 years), (ii) indication for sedation (BMP vs. all other indications), and (iii) use of physical restraint (yes/no). The indication variable was dichotomised as BMP versus all other indications, as subgroup analyses with multiple categories yielded unstable estimates due to limited statistical power. Physical restraint was defined a priori as a procedural variable. Other procedural challenges (e.g. insufficient effect, oversedation, and administration problems) were not included in the multivariable model. Physical restraint was explicitly documented as a discrete event in the clinical records, whereas insufficient effect had to be inferred from narrative chart descriptions and represents a heterogeneous composite outcome. These challenges frequently co-occurred and were highly correlated. Including multiple overlapping procedural variables would have introduced collinearity and reduced model interpretability. Physical restraint was therefore retained as a consistently documented and clinically meaningful marker of procedural escalation.

Adjusted ORs with 95% CIs are reported from the regression model. To account for the relatively small sample size and potential model instability, bootstrapping with 5000 resamples was applied to derive bias-corrected standard errors and confidence intervals. Model fit was assessed using likelihood ratio tests, Nagelkerke’s R^2^, and the Hosmer–Lemeshow test. All analyses were conducted using IBM SPSS Statistics, version 29.

## Results

### Prevalence and indications for sedation

**Fig. 1:**
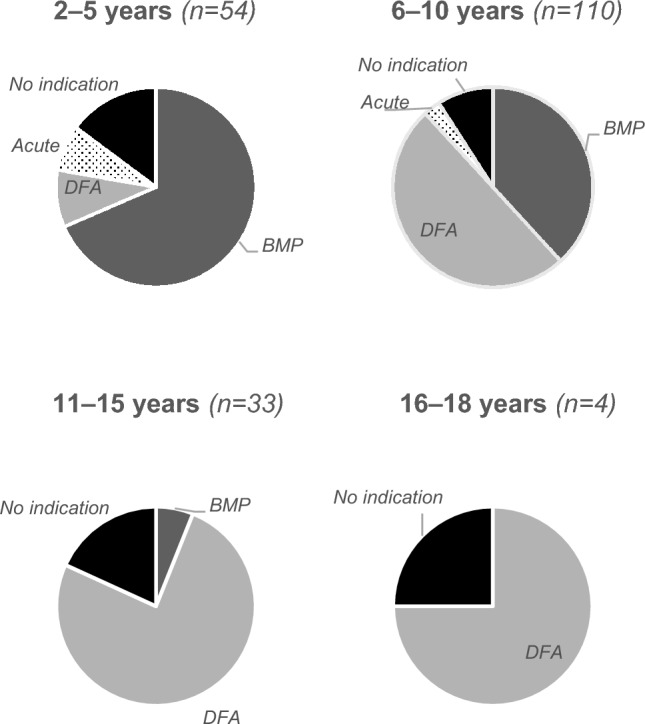
Clinical indications for conscious sedation in paediatric dental care, stratified by age group (total *n* = *201*). Abbreviations: BMP = behavioural management problems; DFA = dental fear/anxiety; acute = acute treatment need; no indication = no indication recorded

During the 12-month period, 201 sedation sessions were recorded across the three clinics. Sedation use varied substantially by age. Most sessions (110; 54.7%) involved children aged 6–10 years, while 0- to 5-year-olds accounted for 54 sessions (26.9%). Only 33 sessions (16.4%) involved 11- to 15-year-olds, and 4 sessions (2%) involved 16- to 18-year-olds. Indications for sedation varied with age (Fig. 1). Among children under 6 years, BMP was the dominant reason for sedation. In contrast, DFA was the main indication among those aged 6–18 years. Indications were not recorded in 12% of sessions.

## Treatment outcomes

Of the 201 sedation sessions, the planned dental procedure was completed in 136 (67.7%), while 65 sessions (32.3%) resulted in dental treatment failure (defined as partial/suboptimal or not performed treatment).

Dental treatment failure was most frequent among the youngest children (0–5 years: 38.9%; 6–10 years: 33.6%; 11–15 years: 21.2%; 16–18 years: 0%). The overall age-related differences were not statistically significant (χ^2^ = 4.92, df = 3, *p* = 0.18).

**Fig. 2:**
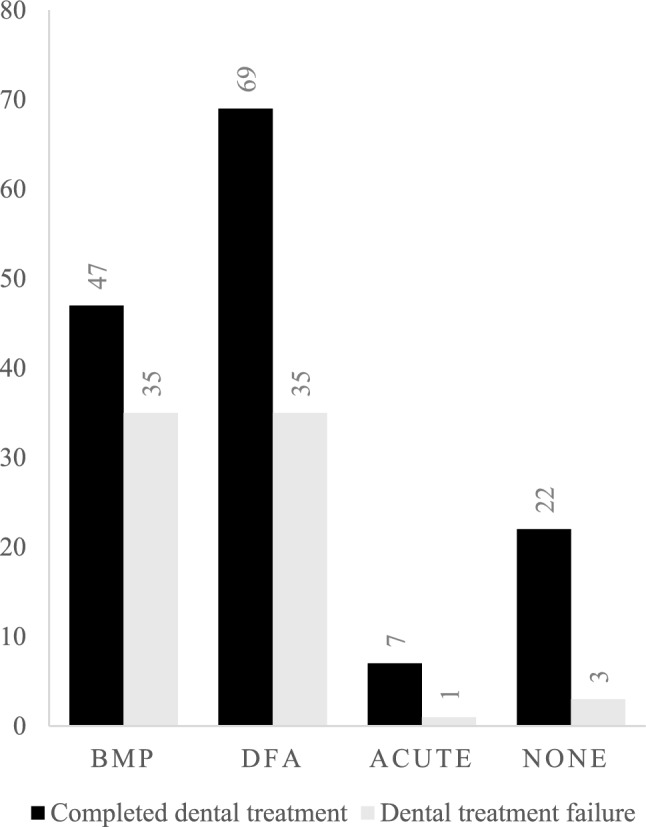
Treatment outcomes across sedation indications. Bars show absolute frequencies to illustrate group size; percentages and statistical comparisons are provided in the text. Abbreviations: BMP = behavioural management problems; DFA = dental fear/anxiety; acute = acute treatment need; no indication = no indication recorded

Figure 2 illustrates treatment outcomes across indication groups. Dental treatment failure did not differ significantly between children with and without DFA as the indication compared with all other indications (33.7% vs. 30.9%, χ^2^ = 0.07, df = 1, *p* = 0.79). In contrast, failure was significantly more common when sedation was indicated due to BMPs compared with all other indications (42.7% vs. 25.2%, χ^2^ = 6.00, df = 1, *p* = 0.014). Sessions recorded without a specific indication had the lowest risk of failure (12.0%, χ^2^ = 4.39, df = 1, *p* = 0.04). Only a small number of sessions were recorded as acute (n = 8), of which seven were completed (12.5%. χ^2^ = 0.70, df = 1, *p* = 0.40).

## Procedural challenges

Most sedation sessions did not report any procedural challenges (*n* = 124, 61.7%). The most frequent problem was an insufficient sedative effect, documented in 40 sessions (19.9%). Physical restraint, defined as holding the child’s head or limbs, was reported in 33 sessions (16.4%), while administration problems (e.g. refusal, spitting out, or partial ingestion of medication) occurred in 18 sessions (9.0%).

Procedural challenges frequently overlapped. In particular, insufficient sedative effect co-occurred with physical restraint in 10 of the 40 sessions (25%) where insufficient effect was recorded.

**Fig. 3:**
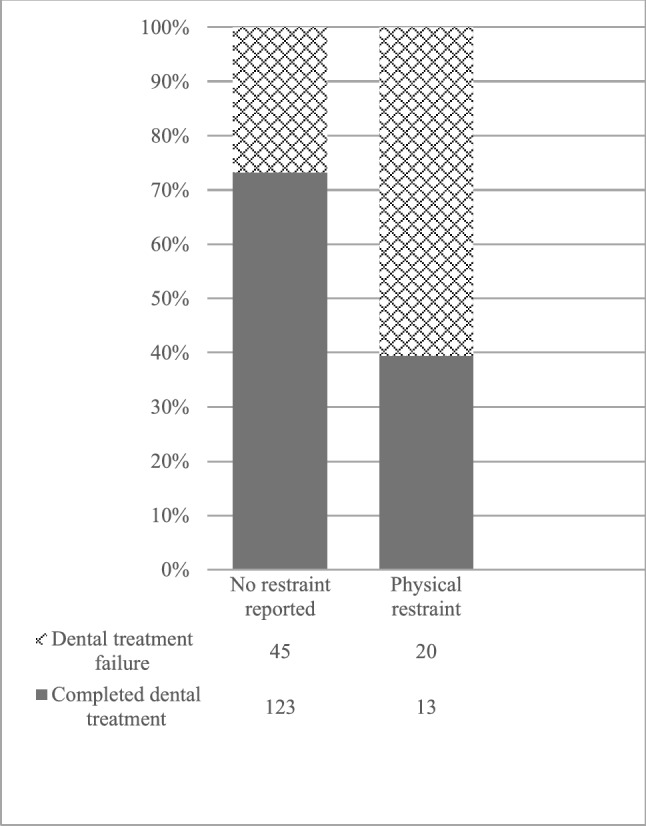
Dental treatment outcomes in sessions with and without documented use of physical restraint

All procedural challenges, except oversedation, were strongly associated with dental treatment failure. Treatment failure occurred in 75.0% of sessions with insufficient sedative effect, compared with 21.7% without (χ^2^ = 39.14, df = 1, *p* < 0.001). Failure was also more frequent in sessions where physical restraint was documented (60.6% vs. 26.8%, χ^2^ = 12.91, df = 1, *p* < 0.001; Fig. 3) and where administration problems occurred (88.9% vs. 26.8%, χ^2^ = 26.13, df = 1, *p* < 0.001). Oversedation, as indicated by oxygen desaturation or reduced consciousness, was rare (*n* = 3, 1.5%); all cases were managed within the clinic without further adverse outcomes, and none were associated with treatment failure.

## Post-operative follow-up

**Fig. 4:**
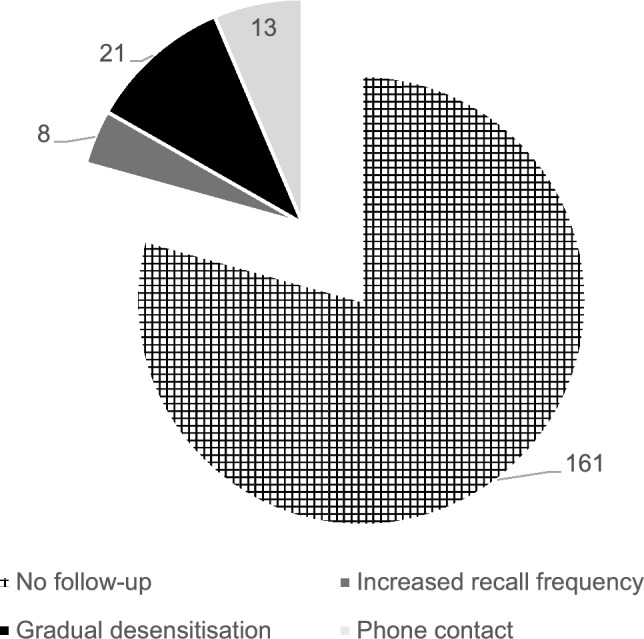
Follow-up after conscious sedation

A follow-up after sedation was documented in only 21% of sessions, meaning the majority (*n* = 161) lacked documentation of any form of follow-up (Fig. 4). Where documented, follow-up most often consisted of a planned recall, sessions with gradual desensitisation, or telephone contact. Although multiple follow-up categories could be coded, this occurred only in two cases (telephone contact combined with either desensitisation or a shorter recall interval).

Among the 65 sessions that resulted in dental treatment failure, follow-up was documented in most but not all cases. In 27 sessions (41.5%), a new attempt with sedation was scheduled within 6 months, and in 8 sessions (12.3%) after a longer interval (> 6 months). Referrals were recorded in 24 cases: 23 to general anaesthesia (35.4%) and 1 to a paediatric dentist (1.5%). Overlap across categories was rare; only two sessions were coded both with a new sedation attempt and referral (to a paediatric dentist or to general anaesthesia). In eight sessions (12.3%), no follow-up of any kind was documented after dental treatment failure.

## Factors associated with dental treatment failure

As treatment failure varied across indications and procedural challenges, we next examined factors associated with failure. Bivariate analyses revealed that both BMP and the use of physical restraint were associated with significantly increased odds of dental treatment failure, whereas the age group showed no significant effect. In the multivariable logistic regression model (Table 1), BMP remained significant (adjusted OR = 1.97, 95% CI [1.01–4.08], *p* = 0.04). Physical restraint was associated with higher odds of treatment failure (adjusted OR = 3.95, 95% CI [1.84–10.22], *p* < 0.001). Age group (11–18 vs. 0–10 years) was not significantly associated with treatment failure (adjusted OR = 0.69, 95% CI [0.22–1.69], *p* = 0.45). The overall model was statistically significant (χ^2^(3) = 19.94, *p* < 0.001), with Nagelkerke’s *R*^2^ = 0.13, indicating modest explanatory power.

**Table 1:** Binary logistic regression examining factors associated with dental treatment failure (*n* = *201*), with bootstrapped standard errors and 95% confidence intervals (*5000 samples*)

Explanatory variable	Crude OR (95% CI)	Adjusted OR (95% CI*)	*p*
Age (11–18 vs. 0–10)	2.35 (0.97–5.67)	0.69 (0.22–1.69)	0.45
Behavioural management problems (yes vs. no)	2.21 (1.21–4.04)	1.97 (1.01–4.08)	0.04
Physical restraint (yes vs. no)	4.21 (2.08–8.54)	3.95 (1.84–10.22)	< 0.001

*Abbreviations* OR = odds ratio; CI = confidence interval. Reference categories: completed treatment (outcome), age 0–10 years, no BMP, no restraint. *Bootstrapped CIs were estimated with 5000 resamples

## Discussion

This audit finds that conscious sedation is not a marginal intervention, but a regularly applied tool in the Norwegian PDS. Approximately, one-third (32%) of sedation sessions resulted in incomplete or compromised treatment. This proportion is higher than that reported in international surveys, which typically find that “inability to complete treatment” accounts for approximately 10% of cases (Coulthard et al. 2015). However, such comparisons must be interpreted cautiously. In the present audit, treatment failure was defined broadly to include partial or suboptimal procedures, whereas survey data typically rely on clinicians’ subjective judgements of completion and provide limited information on procedural complexity.

A further explanation for the observed failure rate relates to the characteristics of the children receiving sedation and the organisational context in which sedation decisions are made. Many children displayed marked behavioural resistance and might, from a clinical perspective, have benefited from more extensive behavioural management, specialist-led care, or general anaesthesia. In the Norwegian PDS, however, access to paediatric dental specialists, inhalation sedation, and general anaesthesia is limited and often centralised. Long travel distances and waiting lists may therefore shape clinical decision-making, increasing reliance on oral sedation even when behavioural resistance suggests a low likelihood of successful treatment.

Understanding treatment failure, therefore, required close attention to how sedation indications were documented and applied in clinical decision-making. In this audit, behavioural management problems emerged as the strongest associated factor of unsuccessful sedation, suggesting that the child’s observable capacity for cooperation is central to treatment outcome. Documentation of dental fear/anxiety (DFA) in electronic records was highly dependent on clinicians explicitly noting fear, as no structured assessment tools or predefined fields were available. As a result, overt or verbalised fear was more likely to be recorded, while subtler or internalised forms of anxiety may have remained undocumented. Consequently, BMP may in some cases reflect unrecognised fear or distress that is expressed behaviourally rather than verbally.

The absence of structured assessment tools also contributed to overlap between DFA and BMP. Although conceptually distinct, DFA involving reported or inferred fear and BMP involving observable resistance, the two often coexist, particularly in younger children. For dental treatment outcomes under conscious sedation, however, the functional impact of behaviour on cooperation appears more decisive than the presumed emotional origin. This helps explain why BMP—but not DFA—was associated with treatment failure in the present analyses. Instruments such as the Indicator of Sedation Need (IOSN) (Coulthard et al. 2011) illustrate how anxiety, medical background, and procedural complexity can be combined to support more consistent case selection, but such tools are not currently implemented or validated in the Norwegian PDS.

The findings also raise ethical concerns. Behavioural resistance can be understood as a form of communication, particularly in children who are unable to articulate fear or discomfort verbally. Sedation may reduce overt distress but does not address the child’s underlying emotional needs and may undermine trust if used in the context of high resistance. In this audit, sedation was least effective when behavioural resistance was high, reflected in the strong association between BMP, physical restraint, and treatment failure. Because data were collected retrospectively, the temporal sequence between insufficient sedative effect, behavioural escalation, and the use of restraint cannot be determined. It is plausible that the use of physical restraint reflects insufficient sedative effect, potentially related to pharmacological factors such as dosing or individual variability in response; however, the present audit focuses on clinical responses when sedation does not achieve its intended effect rather than on underlying pharmacological mechanisms. Physical restraint should therefore be interpreted as a marker of procedural escalation in challenging treatment situations rather than as a causal factor in treatment failure.

In this study, restraint refers to the documented act of physically restricting a child’s movement during treatment. Although terminology varies in the literature, the present audit focuses on this practice rather than on semantic distinctions. In line with European and Norwegian paediatric dental guidelines, physical restraint during conscious sedation is discouraged, as it conflicts with the intended role of sedation as a supportive measure to facilitate coping and cooperation (Hallonsten et al. 2013; Norwegian Directorate of Health 2024). Yet studies show that dentists may legitimise restraint when treatment is deemed necessary (Aarvik et al. 2021), sometimes relying on the amnesic effects of midazolam to justify its use (Lourenço-Matharu and Roberts 2010). The use of midazolam-induced amnesia to justify restraint is problematic, as amnesia does not prevent the encoding of implicit emotional or sensory memory (O’Dwyer et al. 2020; Jafarian et al. 2023; Nyhus and Curran 2012). Experiences involving loss of control or helplessness may therefore contribute to later anxiety or avoidance, even in the absence of explicit recall (Gentsch and Kuehn 2022).

The limited success of sedation in children with marked behavioural resistance highlights the limits of pharmacological support when cooperation cannot be achieved. While clinician competence and training were not assessed in this audit, existing evidence indicates substantial variation in confidence in behavioural management techniques, including among paediatric dental specialists (Wilson and Alcaino 2011). In the Norwegian PDS, where conscious sedation is primarily administered by general practitioners and alternative treatment pathways may be constrained, this underscores the importance of careful case selection and appropriate follow-up when sedation does not achieve the intended outcome.

Although conscious sedation appeared safe in the immediate clinical setting, the lack of systematic follow-up documentation precludes assessment of longer-term biological and psychosocial effects. This represents a critical blind spot, as post-sedation experiences may influence children’s future perceptions of dental care. Sedation practices must also be understood within a broader context of child vulnerability. Social disadvantage and adverse childhood experiences are known to shape both oral health and the ability to cope with dental treatment (Kvist et al. 2018; Myran et al. 2025), yet such factors are rarely documented in routine dental records.

As childhood caries has shifted from a near-universal condition to one concentrated in specific subgroups, the small proportion of children who struggle most with dental treatment require sustained, relationship-based care. Differences across European contexts further contextualise these findings: in several countries, inhalation sedation and specialist-delivered services are more widely available, whereas reliance on oral benzodiazepine sedation is less common. In the Norwegian PDS, uneven access to such alternatives may contribute to the continued use of oral sedation beyond its intended supportive role.

## Strengths and limitations

A key strength of this audit is its use of real-world clinical data from the Norwegian PDS, offering a rare practice-based insight into paediatric sedation as it is documented and applied in everyday care. Unlike surveys, these data reflect routine clinical records rather than retrospective clinician recall, providing a pragmatic perspective on how guideline principles are operationalised in practice.

Several limitations must be acknowledged. Most variables, apart from age and treatment outcome, were derived from free-text clinical notes, introducing potential misclassification, particularly between DFA and BMP. The retrospective design also precluded assessment of the temporal sequence between insufficient sedative effect, behavioural escalation, and the use of physical restraint; restraint is therefore interpreted as a marker of severe behavioural resistance rather than a causal factor in treatment failure. Similarly, the absence of medication and dose variables precluded assessment of pharmacological contributors to insufficient sedative effect, and the audit was therefore not designed to examine pharmacological mechanisms underlying sedation failure.

The dataset did not include sociodemographic or medical information, preventing adjustment for confounding factors such as socioeconomic background, comorbidities, or previous dental experiences. The relatively small number of sedation sessions (*n* = 201) and uneven subgroup sizes, especially among adolescents and acute cases, limited statistical power and widened confidence intervals. Logistic regression should therefore be interpreted as exploratory rather than confirmatory. Bootstrapping with 5000 resamples increased the robustness of estimates but could not compensate for data limitations.

Finally, nitrous oxide inhalation sedation was not included, limiting comparability with settings where combined or inhalation-based sedation is routinely used.

Despite these limitations, the audit provides one of the few empirical snapshots of conscious sedation practices in a publicly funded dental system and highlights key areas for quality improvement and future research.

## Conclusion

Conscious sedation is regularly used in the Norwegian PDS and appears safe in the immediate clinical setting. Nevertheless, approximately one-third of sedation sessions resulted in incomplete or compromised treatment, particularly when behavioural resistance was present. These findings underscore the importance of careful indication and case selection when behavioural management problems are evident.

The absence of structured anxiety assessment and systematic follow-up limits insight into longer-term psychosocial outcomes and may disproportionately affect vulnerable children. Treatment success should therefore be judged not only by procedural completion, but also by whether the child’s experience supports trust, coping, and willingness to engage in future dental care. Overall, the audit highlights structural and documentation gaps that current guidelines now address more explicitly, pointing to the need for more consistent assessment, follow-up, and system-level support.

## Clinical implications

In children with behavioural management problems, sedation was sometimes sufficient to facilitate treatment; however, outcomes were less predictable and failure rates were significantly higher than for other indications. These findings support viewing sedation as an adjunct to behavioural management rather than a stand-alone solution.

Treatment success should be defined not only by procedural completion, but also by whether the child’s experience promotes coping and willingness to return for future care. In situations where referral pathways are limited, dental professionals share responsibility for highlighting system constraints rather than adapting practice in ways that compromise child-centred care.

## System-level implications

Because these data were collected prior to the publication of the current national guideline, the observed variation in documentation and follow-up reflects organisational conditions of that earlier period rather than deviations from an established standard. At the same time, the audit highlights structural areas that the updated guideline now articulates more explicitly.

The absence of standardised documentation for anxiety, behavioural resistance, restraint, and follow-up limited both clinical oversight and quality monitoring. Incorporating structured fields for these elements in electronic records would support more consistent assessment, follow-up, and evaluation of sedation outcomes.

The association between BMP and treatment failure further indicates the importance of access to appropriate referral pathways and alternative treatment modalities. Strengthening system capacity may help ensure that sedation is applied in line with child-centred care principles rather than as a response to unresolved cooperation difficulties.

## Data Availability

The datasets analysed during the current study are not publicly available, as they belong to the county dental services.
